# Exploring the Relationship Between Value Modularity, Knowledge Transfer, and Firm Innovation Performance: Evidence From China

**DOI:** 10.3389/fpsyg.2021.772231

**Published:** 2022-01-06

**Authors:** Jianhua Wang, Yan Zhao, Xiao Han, Luying Li, Samma Faiz Rasool

**Affiliations:** ^1^Dong Fureng Institute of Economic and Social Development, Wuhan University, Wuhan, China; ^2^School of Management, Shanghai University, Shanghai, China; ^3^School of Management and Economics, North China University of Water Resources and Electric Power, Zhengzhou, China; ^4^School of Management, Zhejiang University of Technology, Hangzhou, China

**Keywords:** firm’s innovation performance, knowledge acquisition, knowledge internalization, value modularity, resource integration ability

## Abstract

This study aimed to explore the influence the value modularity and a firm’s innovation performance, directly and indirectly, using knowledge management as mediating variable. Moreover, in this study, we used the resource integration ability as a moderator between the relationship value modularity and firm innovation performance. We collected data from the Chinese state-owned and state-controlled high-tech firms from 2011 to 2018. In this study, we used the gray comprehensive evaluation method to test the degree of value modularity, and hierarchical regression analysis is used to analyze the relationship among variables. The outcomes of this study indicate that value modularity and firm innovation performance has inverted U-shaped and significant association. Similarly, results also confirm that knowledge acquisition and knowledge internalization mediate the relationship between value modularity and firm innovation performance. The finding of this research also confirms that resource integration ability negatively affects the relationship between value modularity and firm innovation performance. This paper enriches the research of the value modularity and gives certain inspiration to knowledge management. At the end of this study, we also suggest some significant practical implications.

## Introduction

Innovation is the driving force of national science and technology development. Similarly, technological innovations are the backbone of firm development. According to the Chinese National Bureau of Statistics survey data, 20.1% of Chinese firms have carried out innovation support. Moreover, from 20.1% the manufacturing and information technology service industry is taking 40% share. Corporate innovation with the participation of multiple subjects has become the main mode of technological breakthrough. Previous studies show that value modules are becoming a trend in the high-tech industries (Gaynor and Bradner, 2001; Zhu, 2003; Bendoly et al., 2004). Besides, innovation is gradually becoming a modularity process, product production, product design, and organization design gradually tend to be modularized. Under the trend of 3c industrial integration in the 1990s, the United States, Japan, and European countries used modularity as a carrier to actively promote the adjustment and development of technology (Zhu, 2003). Modularity can be regarded as an external organization of firms, which can supplement the firm with heterogeneous knowledge (Cheng and Shiu, 2016). Baldwin and Clark (1997) believe that modularity can stimulate innovation, accelerate the rate of industrial change, and change the relationship between firms. Previous studies have also shown that modularity reduces the knowledge correlation between different divisions of labor within the system and promotes the growth of knowledge (Baldwin and Clark, 1997; Ravasz et al., 2018). Based on the SECI model, Nonaka and Tadeuchi (1995) regards the value module as a “ba” for storing knowledge, which promotes the efficiency of knowledge transformation and enhances the knowledge storage of the entire organization. Therefore, Chinese national policies encourage corporate innovation. As a new model of cooperating innovation, value modules have a close relationship with knowledge transmission. So, the management of its structure and internal knowledge transmission is vital to future industrial development. Thus, the literature suggests that value modularity has a positive and significant impact on knowledge transmission and firm innovation.

Prior studies indicate that that resource integration ability plays a positive role in the process of firm innovation (Mele et al., 2010; Pan et al., 2018). Edelman et al. (2010) claim that firm innovation depends on human, technological and financial resources. However, firms often face the problems of lack of innovation resources and low utilization of innovation resources that negatively affect the firm’s innovation behavior (Rasool et al., 2019). Consequently, to ensure the implementation of innovative behaviors, firms inevitably strengthen the absorption, management, and utilization of resources (Wales et al., 2013). Thus, it is believed that resource integration ability is one of the firm abilities to enhance, and few scholars are skeptical about the active utility of resource integration ability. However, from the perspective of bounded rationality, high resource integration ability is not always an incentive for innovation entities. Prospect theory shows the bounded rationality perspective, which thinks subjects often follow the principle of risk aversion rather than the principle of maximizing benefits when they face risks (Rasool et al., 2021). In this theoretical context, strong resource integration ability will weaken the firms’ risk aversion tendency. This is why when subjects face positive feedback, they tend to be “self-good,” and the risk aversion of gains and loss is weakened (Jaworski and Kohli, 1991; Belschak and Hartog, 2010). This means that the incentive effect of resource integration ability is potentially negative, and it is important to reveal the mechanism of its occurrence.

In innovation activities, knowledge flow is an effective transmission mechanism (Dyer and Singh, 1998; Zhou and Wu, 2018). With the help of knowledge flow, the innovation subjects in the corporate organization absorb internal and external knowledge to improve technical abilities. Knowledge acquisition is a positive means of acquiring knowledge from the outside, and many studies have confirmed its positive role. (Inkpen, 1998; Mingliang and Bin, 2008; Zhou and Caroline Bingxin, 2012). However, with the increasing awareness of intellectual property protection, firms will strengthen the risk aversion of the loss of their core resources when facing the threat of knowledge leakage, which may be hindering the process of knowledge acquisition. Knowledge internalization is an important part of the firm learning process in a collaborative context, which is the most critical stage to transform external knowledge into valuable knowledge (Tsai et al., 2016; Wipawayangkool and Teng, 2016). Therefore, knowledge acquisition and knowledge internalization have been widely used to transfer knowledge from top management to support staff. It is important to study knowledge acquisition and knowledge internalization as the path to transmit the influence of the value modularity to firm innovation.

Based on the above debate, very few researchers have investigated the relationship between value modularity and firm innovation performance. In particular, the relationship between value modularity, resource integration ability, knowledge acquisition, knowledge internalization, and firm innovation performance still needs to be researched. Therefore, in this study, first, we investigated the direct relationship between value modularity and firm innovation. Second, we tested the moderating effect of resource integration ability between value modularity and firm innovation performance. Third, we explored the negative relationship of value modularity with knowledge acquisition. Fourth, we verified the knowledge acquisition mediating effect between value modularity and firm innovation performance. Fifth, we checked the mediating effect of knowledge internalization between t value modularity and firm innovation performance. Moreover, in this research, we used the prospect theory and information comparison theory to test the above relationships. However, the comprehensive aims of this study were to explore the process of knowledge flow in value modular and try to reveal the law of decision-making behavior of firms in the process of knowledge transmission, which provides theoretical support for guiding the firm behavior in the value module.

The structure of this study is as follows: Second part explained the theoretical framework and hypotheses development. The third part entailed the details about the research methods of this research. In the fourth part, the analysis and results were explained in detail. The fourth part includes a discussion. The fifth part was describing the conclusion and practical implications of the study. The last part was explaining the limitations and future research directions.

## Theoretical Framework and Hypotheses Development

The study of Kahneman and Tversky (1979) proposed the prospect theory based on the improvement of the classic expected utility theory in psychology. It believes that managers have limited theoretical behavior characteristics, and the performer makes decisions based on the existing experience and maximizes the value of the prospects. The previous studies based on the core content of prospect theory have verified the existence of reference dependence, risk reversal, loss aversion, diminishing sensitivity, and framing effects in the process of user information search (Mansourian and Ford, 2007). There are also studies based on prospect theory, considering the behavior characteristics of participants under limited theoretical conditions, and improving and expanding the dynamic game evolution process under the interaction of government-firm-university institutes (Mindruta, 2013). Based on the risk aversion principle of prospect theory, this article can more realistically analyze the situation when subjects choose strategies that give a reasonable description of the degree of the risk appetite of decision-makers.

According to Aoki (2003) modularity is a complex subsystem composed of semi-autonomous subsystems interconnected with other subsystems and certain rules, reflecting the new industrial structure. Value modularity is a process of resource value re-integration among firms based on specialized knowledge division and core competitive advantages (Zhu, 2003). Prospect theory demonstrates that a firm’s decision-making behavior is based on the psychological measurement of the risk-return relationship, which mainly follows the principle of loss aversion rather than the principle of profit maximization (Ariely et al., 2005; Gattringer et al., 2017). Whether a firm conducts resource exchange oriented by a conservative mentality or actively opening to the outside world is not necessarily linearly related to the intensity of value modularity but is related to the rational measurement process of the relationship between firm investment and risk (Maggi, 2006). Moreover, based on Cremer’s information comparison theory, the process of the firm’s profit-risk relationship can be described from the two perspectives of “information assimilation” and “information alienation” (Cremer, 1990). Through these two processes to prove the process of impact of value modularity on firm innovation performance. “Information assimilation” refers to the process of gradually reducing the heterogeneity of knowledge among firms through knowledge sharing. “Information alienation” refers to the process of a gradual increase in knowledge heterogeneity among firms. The superposition of the two processes is the total effect of value modularity on innovation performance.

### Value Modularity and Innovation Performance

Generally, the value relationship between subjects within the cooperative organization is established, the trust and organizational coordination mechanisms are still in the teething stage, and it is not mature (Gattringer et al., 2017). In this stage, the relationship among subjects in a corporate organization is showing an upward trend. Value module as a corporate organization, firms in value module will increase the intensity and breadth of cooperation to seize the opportunity and focus on in-depth information exchanges to highlight their unique position in the value module (Schilling, 2000). Therefore, information sharing will be deepened, and this process is “information assimilation.” Based on the principle of loss aversion, firms become more willing to cooperate actively and are willing to take the risk of becoming a sunk cost to avoid the loss of possible deeper cooperation (Levy and Levy, 2002). The better mobility of information within the value module increased and keep heterogeneous resources. It is more beneficial for firms to acquire valuable heterogeneous resources from the value module, and the possibility of firm innovativeness can be increased. The effective process of information integration, knowledge heterogeneity among firms is reducing day by day the reduce the firm’s competitive advantage (Cremer, 1990). At this time, knowledge flow among subjects may cause firms to face greater potential losses than rewards. Due to knowledge sharing, the strong dependence among firms increases the loss aversion tendency, so firms can reduce learning-oriented cooperation to protect the loss of core resources. Based on the loss aversion principle, firms will change from information sharing to information defense. Thus, firms start up the process of “information alienation” to enhance the knowledge heterogeneity among firms. In this process, if information sharing will be delayed it will reduce the firm innovation performance. Based on the above literature we summarized that the degree of value modularity deepens, the impact of value modularity on firm innovation performance shows a trend of rising first and then suppressing. Therefore, we proposed below mentioned hypothesis.

**H1:** Value modularity has an inverted U-shaped influence on the firm innovation performance.

### Moderating Effect of Resource Integration Ability

The existing studies demonstrated that firms with strong resource integration ability are easier to discriminate external heterogeneous resources, increasing the effectiveness of resource acquisition and improving resource utilization efficiency (Dyer and Singh, 1998; Bitar and Hafsi, 2007). Although this ability can also strengthen firms to adapt to market changes (Brush et al., 2001). It can reduce the take-off between gains and losses when firms face potential risks. This kind of take-off between gains and losses is risk aversion. As far as this risk aversion is concerned, the stronger the resource integration ability is, the weaker the risk aversion is. In the low-to-medium range, as the degree of information assimilation increases, the potential risks associated with information sharing will also gradually increase. Firms with strong resource integration ability are less risk aversion than those with low resource integration ability. Therefore, firms with strong resource integration ability are more likely to lose core resources and are not conducive to improving firm innovation performance.

From a medium to a high degree, the information assimilation among firms is strong, and the knowledge heterogeneity is weak. The high resource dependence among innovation subjects can enhance the firm’s risk aversion. Based on the risk aversion principle, firms start to adopt the “defense” strategy to protect core resources and increase knowledge heterogeneity. At the time, it is even worse for firms with low resource integration ability. Specifically, the probability of firms getting valuable information from the value module is diminishing at this stage. If the resource integration ability is poor, resource acquisition and utilization efficiency are low, which intensifies the inhibitory impact of the high degree of value modularity on firm innovation performance. In summary, the following hypotheses are proposed:

**H2:** Resource integration ability moderates between the relationship of value modularity and firm innovation performance.

### Mediating Effect of Knowledge Acquisition

Knowledge transmission is the process by which the sender transfers knowledge resources to the receiver in a certain way. Previous studies have shown that knowledge acquisition is an important part of knowledge transmission, firms need to actively seek new knowledge to improve innovation performance (Inkpen, 1998; Singh et al., 2002; Sturgeon, 2002; Gebauer et al., 2012; Zhou and Caroline Bingxin, 2012). Firms can acquire knowledge from outside to enrich their knowledge base and thereby enhance the depth and breadth of their knowledge (Zhou and Caroline Bingxin, 2012). Next, firms can explore new market opportunities based on new knowledge to push innovation.

It has been claimed that knowledge acquisition requires an active approach, firms can openly interact with the business environment in this way, which is conducive to the acquisition of external knowledge (Li and Gao, 2021). Value modularity as this active approach provides a platform for the occurrence of knowledge acquisition (Sturgeon, 2002). In comparison with alliance networks, value modules are more tightly connected among firms. Strong relationships are likely to cause the risk of free-riding behavior or accidental spillover of information in cooperation. Based on the principle of loss aversion, strong relationships induce firms to worry more about the loss of core resources. So, firms will act defensively at this time. Firms may even identify the knowledge acquisition opportunities from external innovation subjects and then initiate knowledge desorption to hinder other learning-oriented firms acquire their core information. Resource capacity of the value module is reduced due to knowledge desorption, which is detrimental to the firms’ acquisition of heterogeneous resources. Therefore, it is failed to the development of innovative activities. In summary, the following hypotheses are proposed:

**H3:** Value modularity has a negative influence on knowledge acquisition.

**H4:** Knowledge acquisition mediates the relationship between value modularity and firm innovation performance.

### Mediating Effect of Knowledge Internalization

Knowledge internalization is used as the measure of knowledge transfer outcomes (Aquino and Castro, 2017). Knowledge internalization is first related to the ability to see value in the transferred knowledge. To understand the knowledge as something efficient and useful for organizational routine; to see the knowledge as valuable is the premise for motivation to learn and then appropriate knowledge. Otherwise, what we see is a ceremonial or formal adoption (Kostova and Roth, 2002) as previously. When heterogeneous resources are effectively utilized, firm innovation performance can be improved.

**FIGURE 1:**
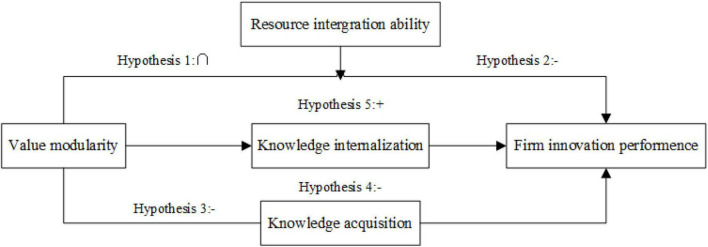
Theoretical Model.

Knowledge internalization is a process by which the innovation subject transforms the externally acquired knowledge into its own explicit or tacit knowledge (Escribano et al., 2009). Knowledge internalization is also seen as a process of searching for valuable knowledge from externally acquired knowledge and transforming and applying it (Goldberg et al., 2006). Value modules have closer linkages than alliance networks, and the heterogeneity of knowledge among firms is higher. Therefore, firms have a greater chance to obtain valuable information from value modules, which would accelerate the process of knowledge internalization. Below mentioned Figure 1 presents the comprehensive theoretical framework of this study. Based on the above discussion, the following hypothesis is proposed:

**H5:** Knowledge internalization mediates between the relationship of value modularity and firm innovation performance.

## Research Methodology

### Data Sources

This paper selects the data of Chinese state-owned and state-controlled high-tech firms from the 30 provinces and municipalities (Tibet was excluded due to incomplete data). With the beginning of a new round of scientific and technological revolution, fierce international competition, and China’s increasing international influence have put forward higher requirements for Chinese industrial structure, leading position, and technological creativity. Chinese state-owned or state-controlled high-tech firms are responsible for major technological breakthroughs in important strategic areas such as manned spaceflight, deep-sea exploration, high-speed railways, domestically produced aircraft, and mobile communications, which are the vanguard of national innovation and development. Among the top 100 firms of the world in 2020, the total number of Chinese firms ranked first, and state-owned firms accounted for 66.71% of the shortlisted Chinese firms. Chinese high-tech firms will face new market opportunities and challenges, value modules as a new industrial structure play an important role in the future international competition (Cheng and Shiu, 2016). Therefore, it is necessary to study the degree of value modularity of state-owned or state-controlled high-tech firms and the process of knowledge flow and value generation within the organization.

The data sources are as follows: first, the data for the evaluation of the value modularity as the independent variables are mainly from China Statistics Yearbook on High Technology Industry and China Torch Statistical Yearbook from 2012 to 2019, and few data comes from the China National Data Information Statistical Website. The evaluation indexes are shown in Table 1. Second, user engagement data was obtained from the Baidu index search of the keyword “state-owned firms,” and the annual average of the Baidu index was selected to measure the user engagement. Finally, the data of dependent variables, independent variables, moderating variables, and control variables were obtained from China Statistics Yearbook on High Technology Industry from 2012 to 2019. The original data were first calculated and then subjected to hierarchical regression analysis.

**TABLE 1:** Evaluation indicators of value modularity.

Module composition	Innovation subject	Level 1	Level 2	Serial number	Unit
Manufacturing value module	Core manufacturing firms; Upstream and downstream firms	R&D status	Number of in-house R&D facilities	*X*_1_	Individual
			New product development projects	*X*_2_	Individual
			Number of patent applications	*X*_3_	Individual
		Element accumulation	R&D full time personnel equivalent	*X*_4_	Individual
			R&D input	*X*_5_	Million yuan
		Collaborative innovation	Percentage of R&D external expenditures	*X*_6_	Million yuan
Integrated value module	Core manufacturing firms	Knowledge sharing level	External knowledge acquisition expenses	*X*_7_	Million yuan
		Level of knowledge alienation	Rate of change of digestion and absorption expenditures versus external technology acquisition expenditures	*X*_8_	Million yuan
Service value module	Government	Government involvement	R&D investment government funding	*X*_9_	Million yuan
Customer value module	Customer	User participation	The annual average value of the Baidu search index for state-owned firms	*X*_10_	/

It is important to better evaluate the organic nature of value modularity. Regarding existing literature, this study divides the value module into four parts, including manufacturing value module, integration value module, service value module, and customer value module. From these four aspects, the process of the resource sharing of the innovation subjects in the value module is evaluated (Adner and Kapoor, 2010; Feng and Wei, 2011) (as shown in Table 1). To obtain the marginal revenue, firms decompose the technical elements in a modular manner. The manufacturing value module mainly includes manufacturing firms such as upstream suppliers. For manufacturing firms, their research and development (R&D) capabilities play an important role. Manufacturing firms need to improve the overall technical level of value modules according to the requirements of integrators (Cui et al., 2014). Therefore, R&D level and factor accumulation are used as evaluation indicators for manufacturing value modules. The integrated value module mainly includes system integrators such as service firms. They concentrate on designing and formulating specific modules, then through cooperation with external firms, the resources of other firms can be used flexibly to obtain competitive advantages. Most of the system integrators are in the core position of value module by paying close attention to the demand trends of consumers, and they stimulate distribution structure, technical requirements, and interface standards of their products. With knowledge as the source of innovation, the level of knowledge sharing and knowledge alienation of integrated firms play a key role in promoting the structural maturity of value modules and accelerating resource reserves. Therefore, knowledge sharing and knowledge alienation are selected as the evaluation indicators of the integrated value module. The service value module and customer value module are measured by government involvement and user involvement, respectively.

### Research Approach

This paper used two research methods. The first was the gray comprehensive evaluation method. It was based on the gray relational theory to evaluate the efficiency of complex systems with complex and incomplete information. Some scholars use the gray comprehensive evaluation method to evaluate an innovation ecosystem; previous scholars use it to evaluate a scientific and technological achievement evaluation system. Since value modularity is an uncertain and dynamically changing complex system, it is more appropriate to evaluate it through the gray comprehensive evaluation method.

First, we established an evaluation index set and select evaluation objects; second, the evaluation index for each object is determined. We supposed that the member of evaluation objects is *m*, and the number of the evaluation indicators of each evaluation object is *n*. *Y_ij_* denotes the best value of the *i* value module at the *j* evaluation index:


$$Y_{ij}\left(i=1,2,\ldots ,m;j=1,2,\ldots ,n\right)$$

Which is:


$$\left[\begin{array}{cc} \begin{array}{ccc} y_{11} & y_{12} & \ldots \\ y_{21} & y_{22} & \ldots \\ \ldots & \ldots & \ldots \\ \begin{array}{c} y_{m1} \end{array} & \begin{array}{c} y_{m2} \end{array} & \begin{array}{c} \ldots \end{array} \end{array} & \begin{array}{c} \begin{array}{c} y_{1n}\\ y_{2n} \end{array}\\ \begin{array}{c} y_{mn}\\ y_{mn} \end{array} \end{array} \end{array}\right]$$

Next, we performed dimensionless processing on the data. $Y_{ij}^{{}^{\prime }}$ is the data after dimensionless processing, *Y*_*max*_ denotes the maximum value in *Y*_*ij*_(*i* = 1, 2, 3,…*m*; *j* = 1, 2, 3,…*n*). The processing method is:


$$Y_{ij}^{{}^{\prime }}=\frac{Y_{ij}}{Y_{max}}$$

Finally, the gray relational degree is calculated:


$$r_{i}=\sum^{n}w_{j}\frac{min_{i}min_{j}\left|Y_{ij}^{{}^{\prime }}-Y_{ij}\right|+\varepsilon max_{i}max_{j}\left|Y_{ij}^{{}^{\prime }}-Y_{ij}\right|}{\varphi_{ij}+\varepsilon max_{i}max_{j}\left|Y_{ij}^{{}^{\prime }}-Y_{ij}\right|}$$

The second method used was hierarchical regression analysis. The hierarchical regression analysis method was to separately analyze and compare two or more regression models, which were usually used in the study of mediation or regulation. The theoretical model constructed in this paper has moderating variables and intermediate variables, so the simple regression analysis model cannot satisfy the verification of the theoretical model in this paper. By using the hierarchical regression analysis method, the main effect, moderating effect, and mediating effect models can be separately analyzed and compared.

### Variable Measurement

The dependent variable was firm innovation performance. The independent variable was value modularity. The moderating variable was resources integration ability. The mediating variables were knowledge acquisition and knowledge internalization. The control variable was the market size, level of R&D investment, and R&D subsidies. All variables and definitions of variables are shown in Table 2.

**TABLE 2:** Variable statistics table.

Variable	Definition
Firm innovation performance	New product sales revenue
Value modularity	The innovation subject in the value module enhances the process of enterprise innovation through resource sharing (As shown in Table 1)
Knowledge acquisition	The ratio of R&D external expenditures to total R&D expenditure
Knowledge internalization	The expenditure on digestion and absorption of technology
Resource integration ability	The rate of change of internal R&D expenditures to external technology acquisition expenditures
Market size	The ratio of main business revenue to the number of firms with R&D investment
R&D investment level	The R&D input of state-owned and state-controlled firms
R&D subsidy	The rate of change in labor costs in R&D internal expenditures

The dependent variable was firm innovation performance. Innovation refers to the innovation of the products sold and the innovation of technology and craftsmanship in the production process (Organisation for Economic Co-operation and Development [OECD], 2007). Innovation performance refers to a quantitative term for the degree of horizontal innovation performance. The patents and new product sales revenue are commonly used by scholars to measure firm innovation performance. In this paper, the new product sales revenue is used as a proxy variable for firm innovation performance. There are two reasons to choose new product sales revenue as the measurement standard: first, despite there being a certain limitation using the sales revenue of new products as the measurement method, a large number of studies have proved their validity as a measure of firm innovation performance. Second, the sales revenue data of new products of China’s state-owned or state-controlled high-tech firms in various regions or municipalities are easier to obtain, while the patent data of each province is less statistically available.

The independent variable was value modularity. Previous Authors pointed out that value modularity is a dynamic and continuous process (Wu and Park, 2009). Managers have gradually realized that achieving strategic mutual trust in an organization is the key to improving the efficiency of resource sharing. The value module as an innovative organization has closer connections among subjects, and the evaluation of value modularity is actually to evaluate the effect of the resources flowing among subjects within the value module. Following previous studies, this paper sorts out the evaluation indicators that are shown in Table 1. The gray evaluation method is used to evaluate the level of the value modularity. The measurement method is as follows:

The paper selected 30 provinces and 10 evaluation indicators. Assuming the set of evaluation indicators is *x* = {*x*_1_, *x*_2_, *x*_3_, … *x*_10_}, and the level of value modularity of the *i* at the *j* evaluation indicator is denoted as *Y*_*ij*_(*x*_*ij*_ = 1, 2, 3, … *n*; *j* = 1, 2, 3, … *m*). In terms of evolutionary time series, the evaluation indicators at different times from 2011 to 2018 constitute eight ecological spaces of 10 × 30, *E* = *x*_*i*_ = {*x*_1_, *x*_2_, *x*_3_, …, *x*_8_}.

Gray correlation degree of value modularity can be calculated by


(1) $$r_{i}=\sum_{j=1}^{n}w_{j}\frac{min_{i}min_{j}\left|Y_{ij}^{{}^{\prime }}-Y_{ij}\right|+\varepsilon max_{i}max_{j}\left|Y_{ij}^{{}^{\prime }}-Y_{ij}\right|}{\varphi_{ij}+\varepsilon max_{i}max_{j}\left|Y_{ij}^{{}^{\prime }}-Y_{ij}\right|}$$

According to the previous calculation methods, in this research, the value of formula (1) is equal to 0.5, the model parameter ε can be derived. Then it is necessary to bring ε into formulation (1) to calculate *r*_*i*_ (*r*_*i*_ as the independent variable “value modularity”).

Moderating variable is resource integration ability, which is expressed as the rate of change of internal R&D expenditures to external technology acquisition expenditures (Zott et al., 2011). Resource integration refers to the ratio of the funds consumed by new knowledge and technology acquired from outside to the funds consumed by using new knowledge and technologies.

Mediating variables are knowledge acquisition and knowledge internalization. Knowledge acquisition is measured by the ratio of R&D external expenditures to total R&D expenditure. Digest and absorb technology expenditure are used as an evaluation indicator of knowledge internalization. In the China Statistics Yearbook on High Technology Industry, the expenditure on digestion and absorption of technology is defined as the cost incurred in the mastery, application, or reproduction of foreign technology.

Three control variables are set. First, there is a constraining or facilitating effect of the high-tech market size on R&D activities (Miotti and Policy, 2003; Miotti and Sachwald, 2003). Generally, if a certain industry has a larger market scale in a region, the innovation investment in that region is correspondingly higher. Vice versa, the innovation investment is low. Based on the consideration of stakeholders, the key influencing factor of market size is selected as the control variable. Market size is measured by the ratio of main business revenue to the number of firms with R&D investment. Second, the R&D investment level is expressed by the R&D input of state-owned and state-controlled high-tech firms. The R&D input varies widely among regions in China and needs to be controlled. The southeastern coastal region has a leading level of economic development and a stronger governmental emphasis on innovation. As a result, industries have richer innovation resources, more sufficient innovation funds to support innovation, and tend to invest more in innovation. In comparison, the northwest has a more backward level of economic development and lower innovation investment. Finally, R&D subsidy is expressed by labor cost than R&D internal funding expenditure. China Statistical Yearbook on High Technology Industry defines R&D internal expenditure as the actual expenditure of the survey unit for internal R&D activities (basic research, applied research, and experimental development). It includes direct expenditures for R&D project activities and indirect expenditures for R&D activities such as management fees, service fees, R&D-related capital construction expenditures, and outsourcing processing fees. Some studies have found that R&D subsidies influence firms’ innovation performance (Dong et al., 2016). Different regions have different levels of financial subsidies, and subsidies are the most direct motivation for firms to innovate. Therefore, it is necessary to control the variable of R&D subsidies in various regions.

### Variable Statistics

**FIGURE 2:**
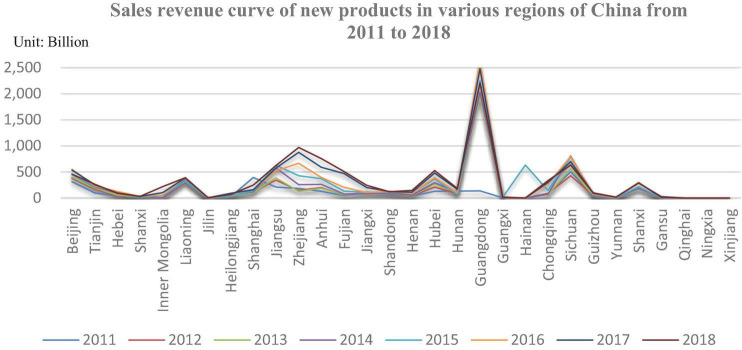
New product sales revenue curve.

**FIGURE 3:**
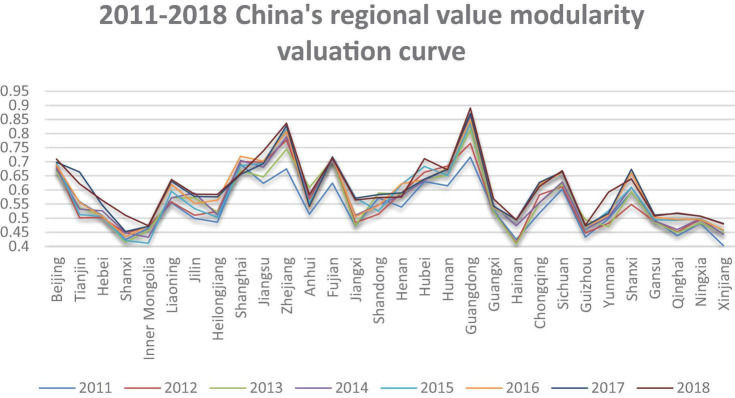
Value modularity evaluation curve.

The data of value modularity is as shown in Table 3. This part visualizes the original data of independent and dependent variables, as shown in Figures 2, 3. These figures show the degree of value modularity and innovation level of state-owned and state-controlled high-tech firms in various regions or municipalities in China. There is a preliminary understanding of the status of cooperative innovation in various regions or municipalities in China.

**TABLE 3:** Value modularity datasheet by regions.

Region	2011	2012	2013	2014	2015	2016	2017	2018
Beijing	0.6691	0.6841	0.6669	0.6758	0.6961	0.6879	0.6983	0.7092
Tianjin	0.5581	0.5018	0.5371	0.5321	0.5128	0.5556	0.6638	0.6221
Hebei	0.5095	0.5024	0.5089	0.5263	0.5062	0.5064	0.5453	0.5628
Shanxi	0.4234	0.4469	0.4139	0.4484	0.4203	0.4345	0.4526	0.51
Inner Mongolia	0.4648	0.471	0.456	0.432	0.4113	0.4716	0.471	0.4732
Liaoning	0.5541	0.557	0.5721	0.5951	0.5951	0.6199	0.6304	0.6367
Jilin	0.4993	0.5103	0.572	0.5888	0.533	0.5524	0.5774	0.5847
Heilingjiang	0.4862	0.5238	0.521	0.5115	0.5033	0.5638	0.575	0.5843
Shanghai	0.691	0.7009	0.6688	0.7049	0.6859	0.7193	0.6549	0.6585
Jiangsu	0.6238	0.6985	0.646	0.6813	0.6953	0.7022	0.693	0.7388
Zhejiang	0.6751	0.7768	0.7449	0.7882	0.8142	0.8086	0.8278	0.8368
Anhui	0.5145	0.5678	0.6096	0.5805	0.5518	0.5359	0.542	0.5837
Fujian	0.6245	0.7091	0.7064	0.7088	0.7134	0.7179	0.7176	0.7135
Jiangxi	0.4728	0.4846	0.4737	0.5104	0.5724	0.5057	0.5711	0.5643
Shandong	0.5718	0.5142	0.5888	0.5469	0.5254	0.5448	0.5833	0.5735
Henan	0.5393	0.5899	0.5855	0.5805	0.6194	0.6217	0.5899	0.5747
Hubei	0.6297	0.6633	0.6383	0.6327	0.6832	0.6366	0.6387	0.7112
Hunan	0.6149	0.685	0.6522	0.6754	0.6519	0.6772	0.6731	0.6717
Guangdong	0.7163	0.7655	0.8141	0.8628	0.8357	0.8495	0.8712	0.8904
Guangxi	0.5275	0.5226	0.5229	0.5453	0.5349	0.5379	0.5424	0.5683
Hainan	0.4243	0.4128	0.4086	0.4732	0.492	0.494	0.4946	0.4934
Chongqing	0.5185	0.5829	0.5554	0.5551	0.6233	0.6204	0.6264	0.6125
Sichuan	0.6034	0.6119	0.6217	0.6286	0.6614	0.6619	0.6644	0.6682
Guizhou	0.433	0.447	0.4948	0.4613	0.4634	0.4688	0.4727	0.4751
Yunnan	0.4979	0.4822	0.4699	0.5037	0.5263	0.5117	0.5175	0.592
Shanxi	0.5923	0.5489	0.5933	0.6096	0.6092	0.6609	0.6733	0.6392
Gansu	0.4933	0.4881	0.4857	0.4901	0.4948	0.5034	0.5111	0.508
Qinghai	0.4375	0.4517	0.4492	0.4986	0.4983	0.4964	0.5162	0.5178
Ningxia	0.4809	0.4959	0.4825	0.4986	0.4983	0.4964	0.5073	0.507
Xinjiang	0.4031	0.4437	0.4492	0.4425	0.4562	0.4582	0.4809	0.4796

Figure 2 shows that the sales revenue curve of new products of state-owned and state-controlled high-tech firms in various regions or municipalities in China from 2011 to 2018. It can be seen from Figure 2 that the sales revenue of new products of state-owned and state-controlled high-tech firms in Guangzhou has been leading the way, and the increase has been the largest in the past few years, from about 1400 billion yuan in 2011 to about 2500 billion yuan in 2019. There is an increase of about 1100 billion Chinese RMB. Besides, the sales revenue of new products of state-owned and state-controlled high-tech firms in Beijing, Shanghai, Liaoning, Jiangsu, Zhejiang, Hubei, and Sichuan is higher than that of other regions or municipalities. This shows that the innovation performance of these regions is high and highlights the innovation vitality and the strong emphasis on innovation by local governments in these regions.

Figure 3 is a curve diagram of evaluation results of the value modularity of state-owned and state-controlled high-tech firms in various regions or municipalities in China from 2011 to 2018. Through the grayness evaluation method, the value range of the measured value modularity is between 0 and 1. It can be seen that the degree of value modularity is still the highest in Guangzhou, even close to 0.9 in 2018. In addition, the degree of value modularity in the five regions of Beijing, Shanghai, Zhejiang, Fujian, and Hebei all exceeded 0.7 in 2018. Therefore, the degree of value modularity in the five regions of Liaoning, Jiangsu, Sichuan, Chongqing, and Shanxi all exceeded 0.6 in 2018. Thus, the following three points can be drawn. First, it can be seen from these data that Guangdong, as a gathering place for promising young people, has the most innovative vitality, and spontaneous cooperative innovation has been stimulated. Second, the cross-regional cooperation and innovation in the Yangtze River Delta are the most prominent. Shanghai, Jiangsu, and Zhejiang all have a high degree of value modularity and a large increase. Finally, in addition to the coastal areas, the inland of Hubei, Sichuan, and Fujian are more prominent in innovation and development and have great development prospects.

## Results and Analysis

### Data Analysis

In this study, Stata 16 (StataCorp, California, CA, United States) was used to conduct descriptive statistics and correlation tests, and the results are shown in Table 4. In general, the absolute value of the correlation coefficient between the variables was <0.7, so the possibility of multicollinearity between the variables was excluded. The correlation coefficients between market size, R&D investment level, and firm innovation performance were 0.721 and 0.861, respectively, both of which were >0.7. It showed that the market size and R&D investment level needed to be controlled for reducing interference items.

**TABLE 4:** Descriptive statistics and correlation analysis of variables.

Variables	Mean	SD	1	2	3	4	5	6	7	8
1 Firm innovation performance	2623302	4336296	1.000							
2 Knowledge acquisition	0.0576	0.1364	–0.055	1.000						
3 Knowledge internalization	976.8	2759.56	0.180	–0.026	1.000					
4 Resource integration ability	487.277	1552.336	0.005	–0.071	–0.054	1.000				
5 Value modularity	0.5770	0.1028	0.631	0.001	0.219	–0.012	1.000			
6 Market size	0.0232	0.0475	0.721	–0.084	0.193	0.011	0.455	1.000		
7 R&D investment level	517570.5	640833.4	0.861	–0.063	0.148	0.111	0.683	0.670	1.000	
8 R&D subsidy	0.2066	0.6785	0.020	0.013	0.062	–0.038	0.064	–0.014	0.001	1.000

Since the data type in this article is panel data, it is usually necessary to perform a unit root test before performing regression analysis to verify whether there are regression traps in the data. If the result coefficient of the unit root test is not significant, then a further first-level difference test or even a second-level difference test is needed. If the secondary difference test coefficient is still not significant, then we believe that the data has a regression trap, and the next regression analysis cannot be carried out.

Augmented Dickey-Fuller (ADF)-Fisher is a recognized and often chosen method for unit root testing. This paper chooses the ADF-Fisher for unit root tests to verify whether the data selected in this paper exists in regression traps. The results of the unit root analysis are shown in Table 5, where we can observe that the test coefficients of all variables are significant, indicating that the unit root test results are excellent, and there is no need for the next difference test to further prove that the data we selected do not have regression traps. In addition, the Kao cointegration test was performed and the ADF value was 1.6469 (*p* < 0.01). Therefore, there are no false regression traps, and regression analysis can be performed.

**TABLE 5:** ADF-Fisher unit root test.

Variables	ADF-Fisher
Firm innovation performance	119.9143***
Knowledge acquisition	122.9929***
Knowledge internalization	229.5232***
Resource integration ability	392.9764***
Value modularity	208.4047***
Market size	66.7216*
R&D investment level	94.0006***
R&D subsidy	99.3604***

***Indicates p < 0.001, ** indicates p < 0.01, * indicates p < 0.05.

### Model Analysis

The data regression results are shown in Tables 6–8. Model 1 is the basic model, which only contains control variables; model 2 adds the primary term of the independent variable based on model 1; model 3 adds the quadratic term of the independent variable and the independent variable to test hypothesis 1; model 13 adds the interaction term of resource integration ability and the independent variable to test hypothesis 2, and it also verifies the moderating effect of resource integration ability; model 4, model 5, model 6, and model 7 are the results of the mediating effect regression of knowledge acquisition, which are used to test hypothesis 3 and hypothesis 4; model 8, model 9, model 10, and model 11 are the results of the mediating effect regression of knowledge internalization, which are used to test hypothesis 5.

**TABLE 6:** Results of main effect analysis.

Variables	Firm innovation performance		
	Model 1	Model 2	Model 3
Market size	2.39*** (6.36)	2.39*** (6.36)	2.10*** (6.19)
R&D investment level	4.642* (6.79)	4.278*** (12.67)	3.993*** (13.03)
R&D subsidy	0.015 (0.78)	0.012 (0.62)	−0.001 (−0.07)
Value modularity		0.331* (1.88)	7.99*** (7.09)
Value modularity^2^			−6.96*** (−7.46)
Constants	−0.365*** (74.18)	0.208** (−2.24)	2.23*** (6.61)
R^2^	0.8281	0.7834	0.8250
F	187.05	212.52	220.66

*** Indicates p < 0.001, ** indicates p < 0.01, * indicates p < 0.05.

**TABLE 7:** Results of intermediary effect analysis.

Variables	Knowledge acquisition		Innovation performance		Knowledge internalization		Innovation performance	
	Model 4	Model 5	Mode6	Model 7	Mode8	Model 9	Model 10	Model 11
Market size	−3.795 (−1.34)	−3.813 (−1.35)	23.092*** (6.19)	20.421*** (6.05)	10.009** (2.01)	10.052*** (2.04)	23.456*** (6.19)	20.599*** (6.02)
R&D investment level	0.001** (2.17)	0.001*** (2.85)	4.738*** (17.04)	4.113*** (13.26)	0.001 (0.38)	−0.001 (−1.11)	4.636*** (16.65)	4.011*** (13.04)
R&D subsidy	−0.09 (−0.61)	−0.066 (−0.45)	0.133 (0.69)	−0.02 (−0.12)	0.261 (1.01)	0.205 (0.80)	0.141 (0.72)	−0.02 (−0.11)
Value modularity		−2.448* (−1.84)		−7.894*** (−7.04)		5.789** (2.49)		−8.036*** (−7.11)
Value modularity^2^				6.841*** (7.37)				69.74*** (7.47)
Knowledge acquisition			−0.002** (−2.45)	−0.0016** (−2.04)				
Knowledge internalization							0.043* (0.88)	0.036* (0.82)
Constants	0.357*** (2.71)	1.626** (2.32)	−0.29* (−1.5)	22.149*** (6.61)	0.618*** (2.66)	−2.382* (−1.94)	−0.391** (−2.20)	22.446*** (6.64)
R^2^	0.021	0.035	0.786	0.828	0.042	0.067	0.781	0.826
F	1.697	2.134	215.3	187.1	3.46	4.203	209.4	183.7

*** Indicates p < 0.001, ** indicates p < 0.01, * indicates p < 0.05.

**TABLE 8:** Results of moderating effect analysis.

Variables	Firm innovation performance	
	Model 12	Model 13
Market size	2.04*** (6.05)	1.98*** (6.24)
R&D investment level	4.113*** (13.26)	4.086*** (13.04)
R&D subsidy	0.02 (−0.12)	−0.012 (−0.07)
Value modularity	7.89*** (7.04)	7.3*** (5.56)
Value modularity ^2^	−6.84*** (−7.37)	−6.35*** (−5.82)
Resource integration ability	−1.57** (−2.04)	3.711 (0.85)
Value modularity × Resource integration ability		−1.359 (−0.87)
Value modularity^2^× Resource integration ability		1.172*** (1.85)
Constants	2.21*** (6.61)	2.04*** (5.26)
R^2^	0.8281	0.8287
F	187.05	139.68

*** Indicates p < 0.001, ** indicates p < 0.01, * indicates p < 0.05.

### Main Effects

According to Haans et al. (2016), firstly, it needs to determine whether the quadratic coefficient of the independent variable is significant. Specifically, it is assumed that the regression equation of value modularity and firm innovation performance is *y* = β_0_ + β_1_*x* + β_2_*x*^2^, *y* represents the dependent variable, *x* represents the independent variable, and β_0_, β_1_, β_2_ are the factory constant term, the first coefficient of the independent variable, and the quadratic coefficient of the independent variable, respectively. It can be seen from model 3 that the quadratic coefficient of value modularity, β_2_ = −6.97, is significant at the 0.001 level. Secondly, it needs to judge the positive or negative *k* when the independent variable takes the maximum and minimum values, respectively. After calculation, *k* = 7.99−13.92*x*. Because the independent variables in this study are standardized by the mean, the *x* is distributed between 0 and 1. When the minimum value of 0 is taken, *k* = 7.99 and *k* are significantly positive. When the maximum value of 1 is taken, *k* = −5.93 and *k* are significantly negative. Finally, it needs to determine whether the inflection point is distributed within the range of the independent variable. The inflection point is −β_1_/2β_2_ = 0.574, which distributes in the range of *x*. Therefore, there is an inverted U-shaped relationship between the value modularity and firm innovation performance, hypothesis 1 is verified.

### Mediating Effect

Table 6 shows the regression results of the mediation effect. Model 4 includes only control variables and it can be seen that the level of R&D investment has a significant positive effect on knowledge acquisition (β = 0.001, *p* < 0.05). From the regression results of model 5, it can be seen that the value modularity has a significant negative effect on knowledge acquisition (β = −2.448, *p* < 0.1), hypothesis 4 is verified. Model 6 shows that knowledge acquisition has a significant negative effect on firm innovation performance (β = −0.002, *p* < 0.05). Model 7 adds knowledge acquisition based on model 3, and the regression results show that the regression coefficient of knowledge acquisition is significant (β = −0.0016, *p* < 0.05), indicating that the negative mediating effect of knowledge acquisition is significant, so there is the occurrence of the process of knowledge desorption. The coefficient of the squared term of the value modularity and the firm innovation performance is significant (β = 6.841, *p* < 0.01). It indicates that knowledge acquisition plays a partially mediating role in the relationship between value modularity and firm innovation performance. Therefore, hypothesis 4 is verified.

Similarly, model 8 only contains control variables, and there is a significant positive effect of market size on knowledge internalization (β = 10.009, *p* < 0.05). From the regression results of model 9 and model 10, it can be seen that the value modularity has a significant positive effect on knowledge internalization (β = 5.789, *p* < 0.05), and knowledge internalization has a significant positive effect on firm innovation performance (β = 0.043, *p* < 0.01). Model 11 adds knowledge internalization based on model 3. The regression results show that the regression coefficient of knowledge internalization and the firm innovation performance is significant (β = 0.036, *p* < 0.01), indicating that the positive mediating effect of knowledge internalization is significant. The coefficient of the squared term of the value modularity and firm innovation performance is significant (β = 69.74, *p* < 0.01), which indicates that knowledge internalization plays a partially mediating role in the relationship between the value modularity and firm innovation performance. Therefore, hypothesis 5 is supported.

As shown in Table 9, it can be seen that the upper and lower limits of the mediating effect of knowledge acquisition do not contain 0 at the 95% CI, and the upper and lower limits of the direct effect of value modularity on firm innovation performance also do not contain 0, which indicates that the mediating role of knowledge acquisition in the path of the relationship between value modularity and firm innovation performance is significant. Thus, hypothesis 4 is further verified. Besides, from Table 10, it can be seen that the intermediary effect of knowledge internalization and the direct effect of value modularity on firm innovation performance do not contain 0 in the upper and lower intervals of the CI, which indicates that the mediating effect of knowledge internalization is significant and hypothesis 5 is further tested.

**TABLE 9:** Results of bootstrap mediated effects analysis (knowledge acquisition).

Index	Effect value	Boot	Boot CI	Boot CI	*z*	Effectiveness ratio	Test results
		Standard error	Lower limit	Upper limit			
Indirect effects	−492428	436385.9	1347729	362872.6	−1.13*	20.49%	Some agents
Direct effect	4579168	2037750	585250.6	8573085	2.25*	10.75%	

According to the critical value table provided by Mackinnon et al. (2002), |z| > 0.9115, p < 0.05, and * in the table indicates significance at the 5% level.

**TABLE 10:** Results of bootstrap mediated effects analysis (knowledge internalization).

Index	Effect value	Boot	Boot CI	Boot CI	*z*	Effectiveness ratio	Test results
		Standard error	Lower limit	Upper limit			
Indirect effects	103020	268507.8	423245.7	629285.6	1.38*	25.2%	Some agents
Direct effect	3983720	1879066	300817.9	7666622	2.12*	25.8%	

According to the critical value table provided by Mackinnon et al. (2002), |z| > 0.9115, p < 0.05, and * in the table indicates significance at the 5% level.

### Moderating Effect

To test the moderating role of resource integration ability between value modularity and firm innovation performance, the regression equation is assumed to be *y* = β_0_ + β_1_*x* + β_2_*x*^2^ + β_3_*xz* + β_4_*x*^2^*z* + β_5_*z* (*z* represents the resource integration ability). According to the study of Haans et al. (2016), the inverted U-shaped curve will become flat if β_4_ is significantly positive, and if β_4_ is significantly negative, the inverted U-shaped curve will become steep. From model 13 in Table 10, β_4_ = 1.172 (*p* < 0.001), which indicates that the moderating effect of resource integration ability makes the inverted U-shaped curve of value modularity and firm innovation performance smooth. Hypothesis 2 is partially tested.

According to the study of Aiken and West (1991), the moderating effect of the inverted U-shaped curve can be tested. When testing the moderating effect of the inverted U-shaped relationship, suppose both the coefficients of “independent variable × moderating variable” and “squared term of independent variable × moderating variable” are significant. In that case, it means that the moderating variable not only changes the shape of the inverted U-shaped but also changes the overall inclination of the curve. The slope of the regression line was calculated for high and low resource integration ability by using the mean value of resource integration ability plus or minus one standard deviation as the grouping criterion. It can be seen from model 13 in Table 10, β_1_ = 7.3, β_2_ = −6.35, β_3_ = −1.359, β_4_ = 1.172, β_1_, β_2_, β_3_, β_4_ are significant, *k* = (2.344*z*−12.7) *x*−1.359*z* + 7.3. It is finally concluded that in the low degree, the simple slope of high resource integration ability is lower than that of low resource integration ability (5.941 < 8.659); in the high degree, the simple slope of high resource integration ability is significantly negative and smaller in absolute value than that of low resource integration ability (6.385 < 10.356), and low resource integration ability reinforces the effect of the value modularity on firm innovation performance. The inhibition of value modularity on firms’ innovation performance. Hypothesis 2 is tested.

**FIGURE 4:**
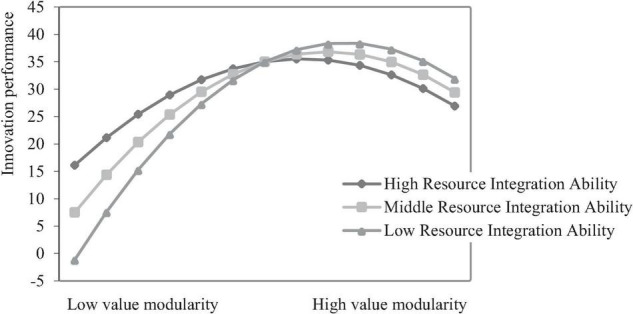
Moderating effect of resource integration ability.

An interactive effect diagram reveals the moderating effect of resource integration ability on the relationship between value modularity and firm innovation performance (as shown in Figure 4). It can be seen from Figure 4 that the curve of high resource integration ability is flatter than that of low resource integration ability, indicating that resource integration ability weakens the inverted U-shaped relationship between the value modularity and firm innovation performance. Before the degree of value modularity reaches the optimal value, firms tend to undergo a process of “information assimilation.” At this stage, compared with low resource integration ability, firms with high resource integration ability can reduce the negative impact for firms. Therefore, high resource integration ability weakens the positive effect of value modularity on the firm innovation performance. Besides, it is increasingly difficult for firms to obtain valuable information in the value module, firms with high resource integration ability can convert marginal knowledge into effective knowledge and implement it than those with low resource integration ability. Thus, high resource integration ability mitigates the inhibitory effect of value modularity on firm innovation performance.

## Discussion

This study selected the data of Chinese state-owned and state-controlled high-tech firms from the 30 provinces and municipalities from 2011 to 2018. Based on prospect theory and information comparison theory, this paper empirically analyzes the relationship among value modularity, knowledge acquisition, knowledge internalization, resource integration ability, and firm innovation performance. This study concludes, first, there is an inverted U-shaped relationship between value modularity and firm innovation performance. Second, as the knowledge transmission path of value modularity on firm innovation performance, knowledge acquisition and knowledge internalization play a mediating role in their relationship. Third, resource integration ability weakens the inverted U-shaped relationship between value modularity and firm innovation performance. Fourth, when the degree of value modularity exceeds a certain limit, it is not conducive to firm knowledge acquisition.

At present, scholars extensively discuss the utility of value modularity. As an organization that is more closely connected and more stable than the network, scholars are interested in what impact it has on the firm innovation (Tao and Xu, 2005). Previous scholars’ research on value modularity is more case studies based on social phenomena, and less empirical research is used to clearly prove the possible relationship between value modules and firm innovation. This study shows that there is a non-linear relationship between value modularity and firm innovation performance, which is an inverted U-shape relationship. The inverted U-shape relationship illustrates that a too high or too low degree of value modularity is not conducive to the improvement of firm innovation performance, while a moderate degree of value modularity is beneficial to firm innovation performance. Before the degree of value modularity reaches the optimal value, “information assimilation” occurs among firms. Firms gradually establish more intimate connections with each other, and active knowledge flow stimulates more innovation activities. However, further information assimilation will increase the risk of core resource leakage. After the degree of value modularity exceeds the optimal value, the heterogeneity of knowledge among firms has become lower due to the occurrence of information assimilation. Based on the risk-averse principle, the risk aversion tendency of firms usually promotes the start of the process of “information alienation,” this process slows down the rate of increase of the knowledge flow path. At this time, path locks and resource locks are formed, which is not conducive to firms’ innovation. This research result provides some theoretical guidance for how to adjust the structure and evolution direction of value modularity in the future.

The role of a complex system is to provide an “efficient place” for the transfer of information, knowledge, and technology among subjects (Sterman, 2010). Knowledge flow often acts as a linker between the system and the subject. Value module as a complex system of multi-agent connection, how to transfer its structural advantages to the firm is a very important problem. The results of this research show that in innovation activities, knowledge acquisition and knowledge internalization serve as effective paths to transmit the influence of value modules to firms. Besides, the degree of value modularity exceeds a certain limit, it is not conducive for firms to obtain knowledge from outside. When the degree of value modularity reaches the optimal value, the relationship among innovation subjects is the closest, and the resource capacity of the value module also reaches the maximum value. After exceeding the optimal value, firms can initiate “knowledge desorption” to protect core resources, which hinder knowledge acquisition among innovation subjects. This research result breaks people’s inherent cognition that a close and complex system may not be able to better promote the flow of information among firms. When the degree of value modularity exceeds a certain level, it will cause the subject’s awareness of information protection. This result will have a deeper inspiration for how to adjust the structure of value modular.

Firm resource integration ability has been proven by scholars to be an important ability for the firm to integrate and filter valuable information from externally acquired knowledge. In the process of knowledge flow in a complex system, the moderating effect of firm resource integration ability will play an important role. In today’s “cooperation and win-win” situation, the positive incentive orientation of resource integration ability is increasingly regarded as a golden rule. However, there are also negative effects of resource integration ability verified. The research results show that under the specific system scenario of value modularity, resource integration ability weakens the inverted U-shaped relationship between value modularity and firm innovation performance. In the low degree of value modularity, firms with high resource integration ability have strong market resilience. From the perspective of bounded rationality, this ability will weaken the firm’s risk aversion and lead to being “self-good” when firms make the decision. This situation usually causes firms to pay insufficient attention to information sharing, which is not conducive to the expansion of knowledge transmission paths among firms. This result is very valuable for us to understand the negative effects of resource integration capabilities, as well as some inspiration for how companies can improve their resource integration capabilities.

## Conclusion, Limitations, and Future Research Directions

### Conclusion

This study aims to explore the influence of the relationship between value modularity and a firm’s innovation performance. In this study, we also investigate knowledge management as mediating variable. Moreover, in this study, we used the resource integration ability as a moderator between the relationship value modularity and firm innovation performance. The outcomes of this research concluded. Firstly, this research enriches the study on the value module’s structural characteristics and knowledge characteristics and opens up the “black box” of knowledge transmission in the value module. This study makes certain contributions to the empirical study on value modularity. Secondly, it clarifies the positive factors and negative factors affecting the knowledge transmission among firms in the value module. This paper made a certain contribution to the development of knowledge management theory in current organizations. Finally, it reveals the negative impact of resource integration ability, which broadens the research on resource integration ability. This paper enriches the research on the application of psychological theory in management.

This research has three outstanding practical values. Firstly, this paper guides the decision-making behavior of firms in the value module, the relationship among firms of value modules should not be too close. Secondly, the role of resource integration ability is better under the high degree of value modularity than at the low degree of value modularity. Firms’ resource integration ability at a high degree of value modularity can alleviate the negative impact of value nodularity on firm innovation by improving resource integration ability. Finally, while actively communicating and cooperating among firms, they need to stay alert to prevent the occurrence of “knowledge desorption.”

There are some recommendations to maintain the moderate degree of value modularity to avoid low knowledge heterogeneity due to excessive knowledge flow. In the low degree of value modularity, like Qinghai, Gansu, etc. Firms in these regions should vigorously open up their boundaries and then actively establish knowledge transfer paths with the outside to improve information sharing. In the high degree of value modularity, firms should recruit partners with heterogeneous resources to gradually expand the scale of value modules and increase the storage of resources. Only by not standing still the value modularity can continue to give full play to the advantage of the platform for continuing to promote firms’ innovation. In addition, it is necessary to adjust the structure of value modules in time and regulate the degree of value modularity in various regions at a moderate degree, in this way to maximize the leverage of value modules and activate the innovation vitality of innovation subjects.

On the whole, from the evaluation results of value modularity, the degree of value modularity in various regions of China is relatively low. Faced with the era that is increasingly inclined to individualization, the production or design process of the product evolves from the “waterfall paradigm→itinerant paradigm→object-oriented paradigm,” value module as an important carrier for the application of object-oriented paradigm can meet the needs of customers for custom-made and efficiency (Zhu, 2003). These modules can produce personalized products according to different customer needs. To meet the needs of the times, value modularity will be the main form of industrial integration and development in the future, so local governments need to strengthen their emphasis on local value modules and increase investment in management, capital, and talent. Thereby breaking through the phenomenon of “fusion of corpses.”

Firms need to pay attention to the problem of “knowledge desorption” initiated from the increased degree of value modularity. This paper advises that in the high degree of value modularity, firms need to look for new opportunities to broaden the boundaries of cooperation or establish new project partnerships to prevent the occurrence of “knowledge desorption.” At the same time, the organizational trust mechanism should be further developed to a certain extent so that the “defense” barrier of firms can be broken down. It also encourages them to open up their boundaries again and actively cooperate with partner firms for innovation. As a measure of the result of knowledge transfer results, knowledge internalization is the key process of knowledge transfer and the key process of knowledge transmission. Firms need to enhance the ability to identify valuable knowledge to accelerate the rate of knowledge internalization.

Under certain circumstances, resource integration ability is a “double-edged sword”, and business managers should pay attention to the dual effects of resource integration ability. At the early stage of the value modularity formation, firms with strong abilities need to eliminate the “self-good” and actively carry out effective innovation cooperation to share information. After the critical value is exceeded, the structure of value modularity is more mature, and firms need to focus on improving their resource integration ability, which helps select valuable information from marginalized resources and weakens the inhibitory effect of the value module on firm innovation performance.

### Limitations and Future Research

One limitation of our study is that the evaluation indicators for value modularity are not comprehensive enough and need to be further improved in the subsequent studies. The paper measures value modularity from four parts: manufacturing value module, integrated module, service value module, and customer module. Manufacturing value module measured by its R&D ability; integrated module measured by the level of knowledge sharing and knowledge alienation between it and other firms; service value module, and customer module is only measured by one index, respectively. These indicators used to measure value modularity are limited. For example, in addition to the support of R&D funds, the government is also the role of market regulation in China, so scholars can find suitable measurement methods to measure it in the future. For the gray comprehensive evaluation method, the more and the more complete the index, the higher the accuracy of the measurement. Therefore, scholars can find more suitable indicators to measure value modularity in the future.

Second, resource integration ability can be specifically divided into four levels: resource identification ability, resource acquisition ability, resource allocation ability, and resource utilization ability. The paper selects resource integration ability as only one moderator and does not start from the four subdivision levels. There may be more interesting results to test the moderating effect from four levels, and this is possibility warrants investigation in future research.

Third, the appropriateness of a control mechanism is likely to depend on the context. Our findings suggest an inverted U-shape relationship between the value modularity and firm innovation performance. These findings apply only to Chinese state-owned or state-controlled high-tech firms and other similar settings. A different pattern of findings may emerge in other contexts. It is therefore vital to examine the efficacy systematically across different firm contexts.

## Data Availability Statement

The raw data supporting the conclusions of this article will be made available by the authors, without undue reservation.

## Author Contributions

JW contributed in the research formulation and conceptualization. YZ supervised and extensively revised this research manuscript. XH participated in the data curation and visualization. LL participated in the conceptualization and actively drafted the manuscript. SR participated in the formal analysis, data interpretation, and revised the original draft. All authors contributed to the article and approved the submitted version.

## Conflict of Interest

The authors declare that the research was conducted in the absence of any commercial or financial relationships that could be construed as a potential conflict of interest.

## Publisher’s Note

All claims expressed in this article are solely those of the authors and do not necessarily represent those of their affiliated organizations, or those of the publisher, the editors and the reviewers. Any product that may be evaluated in this article, or claim that may be made by its manufacturer, is not guaranteed or endorsed by the publisher.
